# Prevalence of fever of unidentified aetiology in East African adolescents and adults: a systematic review and meta-analysis

**DOI:** 10.1186/s40249-023-01105-z

**Published:** 2023-05-25

**Authors:** Faisal Nooh, Afona Chernet, Klaus Reither, James Okuma, Norbert W. Brattig, Jürg Utzinger, Nicole Probst-Hensch, Daniel H. Paris, Anou Dreyfus

**Affiliations:** 1 Swiss Tropical and Public Health Institute, Kreuzstrasse 2, 4123 Allschwil, Switzerland; 2 University of Basel, Basel, Switzerland; 3 College of Medicine and Health Sciences, Jigjiga University, Jigjiga, Ethiopia; 4 College of Medicine and Health Sciences, University of Hargeisa, Hargeisa, Somaliland; 5 Department Infectious Disease Epidemiology, Bernhard Nocht Institute for Tropical Medicine, Hamburg, Germany; 6 Section of Epidemiology, University of Zürich, Zurich, Switzerland

**Keywords:** Aetiology, East Africa, Febrile illness, Fever, Meta-analysis, Prevalence, Systematic review

## Abstract

**Background:**

Primary health care settings and hospitals of low- and middle-income countries have few accessible diagnostic tools and limited laboratory and human resources capacity to identify multiple pathogens with high accuracy. In addition, there is a paucity of information on fever and its underlying aetiology in the adolescent and adult population in East Africa. The purpose of this study was to estimate the pooled prevalence of fever of unidentified aetiology among adolescent and adult febrile patients seeking health care in East Africa.

**Methods:**

We pursued a systematic review using readily available electronic databases (i.e. PubMed, Cumulative Index to Nursing & Allied Health Literature, Scopus, Cochrane Library and Web of Science) without language restriction from inception date of the respective databases to October 31, 2022. We adhered to the Preferred Reporting Items for Systematic Reviews and Meta-Analyses guidelines. Identified studies were screened for relevance. Further analyses based on pre-set eligibility criteria were carried out for final inclusion. Two reviewers independently screened and extracted data. Risk of study bias was assessed. Meta-analysis of the prevalence of fever of unidentified aetiology was performed.

**Results:**

We identified 14,029 articles of which 25 were eligible for inclusion, reporting data from 8538 participants. The pooled prevalence of febrile cases with unidentified aetiology was 64% [95% confidence interval (*CI*): 51–77%, *I*^2^ = 99.6%] among febrile adolescents and adults in East Africa. For the proportion of patients with identified aetiology, the studies documented bacterial pathogens (human bloodstream infections), bacterial zoonotic pathogens and arboviruses as the main non-malarial causative agents in East Africa.

**Conclusions:**

Our study provides evidence that almost two-thirds of adolescent and adult febrile patients attending health care facilities in East Africa might receive inappropriate treatments due to unidentified potential life-threatening fever aetiology. Hence, we call for a comprehensive fever syndromic surveillance to broaden a consequential differential diagnosis of syndromic fever and to considerably improve the course of patients’ disease and treatment outcomes.

**Supplementary Information:**

The online version contains supplementary material available at 10.1186/s40249-023-01105-z.

## Background

Fever is the temporary elevation in body temperature in response to a disease or illness [1, 2]. It is the cardinal sign for an acute infection [3]. Fever is also one of the most common complaints of patients seeking care at hospitals and primary health care settings in low- and middle-income countries (LMICs) [4, 5]. The burden of febrile illnesses is usually estimated based on the identified aetiology. In recent years, attempts were made to address the combined burden of fever-characterised conditions [6]. Crump and Kirk [6] proposed a syndromic approach to all febrile illnesses to enable the assignment of disability-adjusted life years (DALYs) and deaths to specific aetiologic agents.

In primary health care settings of LMICs, fever poses a potential diagnostic challenge. Approximately one-third to half of patients present with fever [7]. Children and seriously ill patients make up the highest percentage of people affected by fever. Importantly, case fatality ratio among patients with fever requiring hospital admission may exceed 20% [8–10] and accurate determination of the underlying cause of fever is challenging due to the wide spectrum of fever aetiologies, the lack of differential diagnostic tools and limitations in access to care and human resources.

Notwithstanding the paucity of fever studies in adults in LMICs, the presence of infections other than malaria underscore the need for evidence-based algorithms to help clinicians manage febrile illnesses [11]. Despite the progress made with analysis by multiplex polymerase chain reaction (PCR) that increases the recognition of multiple possible aetiologies of pathogen-initiated fever [12], hospitals in East Africa currently still have few accessible diagnostic tools and limited laboratory capacity to identify multiple pathogens with high accuracy [13]. It follows that diagnosis of fever predominantly relies on single-disease based investigation in East Africa. As a result, clinicians rely on non-specific clinical data to judge empirical therapy.

The purpose of this paper was to determine the extent of reported fever cases with unidentified aetiologies in adolescents and adults (i.e. individuals aged ≥ 13 years) in East Africa. We pursued a systematic review and meta-analysis to establish an evidence-base for an appropriate fever case management in East Africa. Additionally, our findings should help policy-makers to prioritise healthcare resources and funding towards programmes that strengthen surveillance-response systems to better address fever of unidentified aetiology.

## Methods

We pursued a systematic review and meta-analysis to estimate the pooled prevalence of fever of unidentified aetiology in adolescents and adults in East Africa, following the Preferred Reporting Items for Systematic Reviews and Meta-Analyses (PRISMA) guidelines [14]. We also determined whether patient characteristics, study setting and study design contribute to the observed prevalence of fever of unidentified aetiology in the included studies.

In the context of this paper, we use the term “fever of unidentified aetiology” to describe any documented febrile illness with no identified aetiologic agent and no limitation of fever duration. Of note, this term is distinct from “fever of unknown origin” (FUO) defined as a febrile illness that did not resolve, and with no obvious source despite full investigation, persisting for more than 3 weeks [15].

According to the African Union (AU), Africa comprises of five geographic sub-regions, namely: (i) Central Africa; (ii) Eastern Africa; (iii) Northern Africa; (iv) Southern Africa; and (v) Western Africa [16]. We focus on Eastern Africa (also called East Africa) that consists of the following 14 countries: Comoros, Djibouti, Eritrea, Ethiopia, Kenya, Madagascar, Mauritius, Rwanda, Seychelles, Somalia, South Sudan, Sudan, Tanzania and Uganda.

### Eligibility criteria

We included studies that reported undifferentiated fever (UDF) among febrile patients that met the following criteria. First, studies that reported the proportion of UDF among adolescent and adult febrile patients (aged ≥ 13 years) from health facilities and community-based programmes. Second, UDF studies that were conducted in East Africa. Third, primary UDF studies of both observational and interventional designs (longitudinal and cross-sectional, randomised and non-randomised community trials, controlled and uncontrolled before/after studies).

Any study that met at least one of the following exclusion criteria was removed:

(i) Studies that evaluated patients with a focus on a single aetiologic agent. The focus of this meta-analysis was on fever with unidentified aetiology, and studies that only investigated a specific aetiologic agent would not contribute to the overall understanding of the topic.
(ii) Editorials, reviews, policy statements, case reports, case series studies, perspectives and author replies. These types of studies were excluded because they are not original research studies and do not provide new data that would contribute to the overall analysis. Including them would introduce bias and could potentially skew the results.
(iii) Studies on fever associated with malignancies, autoimmune disorders and immunodeficiency. These studies were excluded because fever in these conditions has a known aetiology, and hence, including them would not contribute to the understanding of fever with unknown aetiology.

### Data sources

We systematically searched the following readily available electronic databases: PubMed, Cumulative Index to Nursing & Allied Health Literature (CINAHL), Scopus, Cochrane Library and Web of Science. We searched from inception to October 31, 2022, without language restriction. The reference lists of relevant studies were hand-searched for additional studies.

### Search strategy

The following strings of word combinations were employed to identify relevant studies:

1. “Fever” OR “fever of unknown origin” OR “FUO” OR “febrile” OR “fever without apparent source” OR “FWAS” OR “undifferentiated fever” OR “febrile state” OR “hyperthermia” OR “pyrexia” OR “febrile syndrome*” OR “fever without source” OR “FWS” OR “fever without a source” OR “acute undifferentiated fever” OR “AUF” OR “acute febrile illness*” OR “undifferentiated fever”.
2. “Diagnosis” OR “diagnostic*” OR “screening” OR “test*” OR “management” OR “clinical”.
3. “Comoros” OR “Djibouti” OR “East* Africa” OR “Eritrea” OR “Ethiopia” OR “Horn of Africa” OR “Kenya” OR “Madagascar” OR “Mauritius” OR “Rwanda” OR “Seychelles” OR “Somalia” OR “South Sudan” OR “sub-Sahara*” OR “Sudan” OR “Tanzania” OR “Uganda”.

### Study records

We screened the identified items and assessed for inclusion. Retrieved studies were transferred to the bibliographic software EndNote™ X9 (Clarivate Analytics; Philadelphia, USA), screened for relevance and checked for duplication. The criterion for relevance was based on the scope and objective of our review. Further analyses based on the eligibility criteria identified the relevant documents for final inclusion. Key data from the included studies were extracted using Microsoft^®^ Excel 2016 (Microsoft; Washington, USA). The retrieved data included: study characteristics, participant characteristics and major findings.

### Risk of bias in individual studies

Two authors (FN and AC) independently assessed and rated the risk of bias on individual studies, following the Joana Briggs Institute (JBI) critical appraisal tool for prevalence studies [17]. Disagreements between the two reviewers were discussed and, if need be, a third author was consulted until consensus was reached.

### Data synthesis

For the included studies, descriptive findings were summarised in tables accompanied by text. We explored the types of diagnostic tests commonly reported by the studies and the aetiologic agents that were identified. We documented the extent of the diagnostic tests performed in each study before any clinical decision was made.

We performed descriptive tasks including comparisons of the studies and patient characteristics. We used metaprop package in STATA version 16 (StataCorp; Texas, USA) to calculate the pooled prevalence of the reported fever of unidentified aetiology [18]. We calculated weighted country-specific and overall pooled prevalence from a random-effects model using inverse-variance weights. The study-specific 95% confidence interval (*CI*) was computed using the exact method. We applied the Freeman-Tukey double arcsine transformation to correct extreme values.

## Results

### Description of search results

**Fig. 1:**
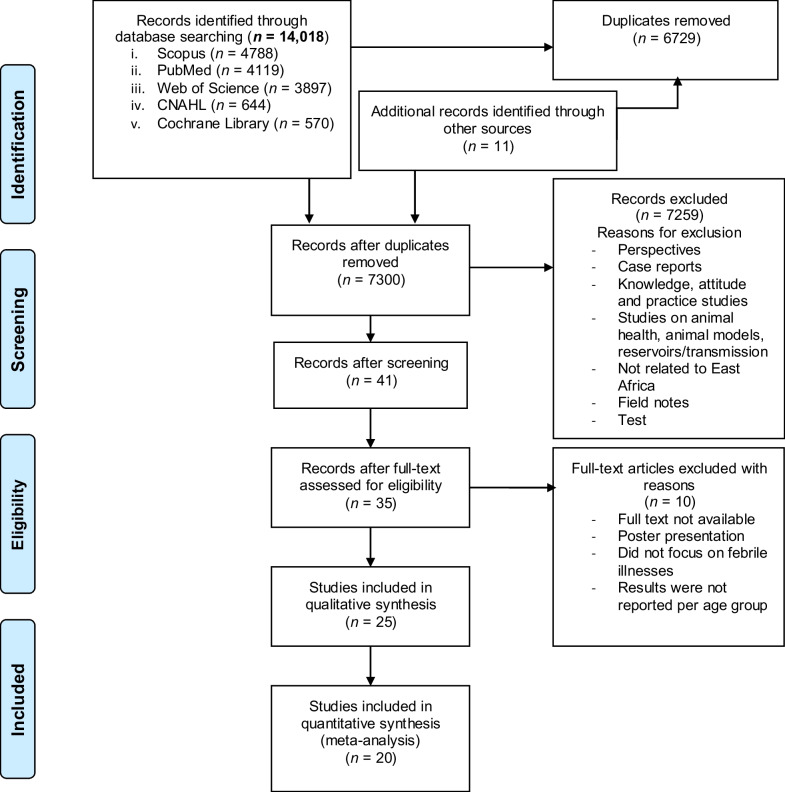
PRISMA flowchart of the available published documents for the prevalence of undiagnosed undifferentiated fever in East African adolescents and adults (aged ≥ 13)

The first database search identified a total of 15,579 items. Scopus (35.5%) and Web of Science (28.5%) contributed the majority of items. PubMed added another 26.4%. We reduced the search results to 14,018 articles after exclusion of reviews. We then imported the refined results into EndNote. Among these articles, Scopus contributed 30.7%, while PubMed and Web of Science contributed 26.4% and 25.0%, respectively (Fig. 1).

Additional searches (e.g. hand searching reference lists of relevant documents) identified another 11 articles (including two items from Google scholar). A total of 6729 duplicates were removed and another 7259 Items were excluded while screening titles and abstracts. As a result, full texts of 35 documents were assessed. Risk of bias was evaluated in these 35 articles. Ten of these articles (29.4%) were excluded due to high risk of bias (Additional file 1: Table S1). Hence, 25 records were retained for qualitative analysis. Overall, 20 studies were included for meta-analysis (Fig. 1).

### Description of studies

The 25 records included into the qualitative synthesis [8, 19–42] reported data from studies conducted in six East African countries: Tanzania (*n* = 15), Ethiopia (*n* = 5), Kenya (*n* = 2), Madagascar, South Sudan and Uganda (*n* = 1 each). The studies were all published in English between 1988 and October 2022. Four articles reported findings from one study of 403 patients in a single hospital [21, 23, 25, 29]. We combined the findings of these articles into a single study. Similarly, another four articles [26, 27, 34, 41] reported results from two studies. Hence, the 25 articles reviewed here reported findings from 20 different studies. Of these studies, six were community- or population-based prevalence studies [22, 32, 35, 37, 39, 40], one study was conducted in a primary health care centre [31] and the remaining 13 studies were hospital-based [8, 19, 20, 24, 27–30, 33, 36, 38, 41, 42]. From the non-hospital based studies, we extracted data reported from febrile individuals only. The duration of data collection varied from one month to more than a year. None of the studies followed patients longitudinally.

Overall, the included studies reported data from 8538 participants, of these 5045 were from hospital and 3493 were from non-hospital-based settings. Few studies enrolled children but described results from children and adults separately. The number of patients in each study ranged from 90 [38] to 1425 [8]. Data extracted from the included studies were obtained from participants aged 13 years and above. Table 1 summarises the characteristics of the 20 included studies.

**Table 1:** Characteristics of 20 included studies

References	Study design and setting	Recruitment period (months)	Year of data collection	Country	Number of patients	Age (years)	Number of patients with unidentified aetiology of fever (%)	Aetiologic agents identified (tested positive)
Zenebe et al. 2011 [24]	Cross-sectional, hospital-based	5	2009/2010	Ethiopia	260	18–61	237 (91.2%)	*Coagulase negative staphylococci (CoNS); Staphylococcus aureus; Escherichia coli; Klebsiella pneumoniae*
Guillebaud et al. 2018 [35]	Cross-sectional, community-based	12	2014/2015	Madagascar	253	≥ 15	167 (66.0%)	*Plasmodium* spp.*; Mycobacterium tuberculosis*; *Leptospira* spp.*; *Epstein-Barr virus; Hepatitis B virus (HBV); Human rhinovirus
Ali et al. 2020 [38]	Cross-sectional, hospital-based	2	2015	Tanzania	90	18–70	48 (53.3%)	Dengue virus*; Brucella* spp*.; Rickettsia* spp*.*
Archibald et al. 1998 [8]	Cross-sectional, hospital-based	2	1995	Tanzania	1425	> 15	1302 (91.4%)	*S. aureus;* non-typhi *Salmonella* spp.*; Streptococcus pneumoniae; Cryptococcus neoformans; M. tuberculosis*
Crump et al. 2011 [21]	Cross-sectional, hospital-based	1	2007/2008	Tanzania	403	> 13	134 (33.2%)	*Salmonella* serotype typhi*; S. pneumoniae; C. neoformans; M. tuberculosis; E. coli*
Crump et al. 2013 [29]	Cross-sectional, hospital-based	12	2007/2008	Tanzania	403	> 13	134 (33.2%)	Chikungunya virus*; Rickettsia* spp.*; Leptospira* spp.*;* spotted fever group (SFG) *Rickettsia spp.; Coxiella burnetii; Brucella* spp.*;* fungus
Felekel et al. 2015 [31]	Cross-sectional, health centre	1	2011	Ethiopia	215	> 16	96 (44.7%)	*Salmonella* serotype typhi*;* Typhus group (TG) *Rickettsia spp.; Borrelia* spp.
Hertz et al. 2012 [25]	Cross-sectional, hospital-based	12	2007/2008	Tanzania	403	> 13	134 (33.2%)	Dengue virus; Chikungunya virus
Mease et al. 2011 [22]	Cross-sectional, population-based	2	2004	Kenya	1,141	> 18	513 (44.9%)	Chikungunya virus*;* Dengue virus*;* West Nile virus*;* Yellow fever virus
Meremo et al. 2012 [27]	Cross-sectional, hospital-based	6	2011	Tanzania	346	> 18	313 (90.5%)	*Salmonella* spp.*; E. coli; S. pneumoniae; S. aureus*
Meremo et al. 2012 [26]	Cross-sectional, hospital-based	6	2011	Tanzania	346	> 18	313 (90.5%)	*M. tuberculosis*; Human immunodeficiency virus (HIV)
Moon et al. 2014 [30]	Cross-sectional, hospital-based	4	2011/2012	Tanzania	193	≥ 13	174 (90.0%)	*Plasmodium* spp.; bacteria
Nadjm et al. 2012 [28]	Cross-sectional, hospital-based	6	2007	Tanzania	198	> 13	137 (69.2%)	Non-typhi *Salmonella* spp.*; Streptococcus pyogenes; E. coli*
Hercik et al. 2018 [36]	Cross-sectional, hospital-based	1	2014	Tanzania	135	≥ 15	97 (28.2%)	*Plasmodium* spp.; *Leptospira* spp.; Dengue virus; *Haemophilus influenzae*; Respiratory syncytial virus (RSV); human Rhinovirus; Adenovirus
Ochieng et al. 2015 [32]	Cross-sectional – population-based	NA	2007	Kenya	1,091	15–64	881 (80.8%)	Dengue virus; Rift Valley fever virus; Chikungunya virus
Prabhu et al. 2011 [23]	Cross-sectional, hospital-based	12	2007/2008	Tanzania	403	> 13	287 (71.2%)	SFG *Rickettsia;* TG *Rickettsia; C. burnetii*
Ssali et al. 1998 [20]	Cross-sectional, hospital-based	4	1997	Uganda	299	15–64	234 (78.3%)	*M. tuberculosis; S. pneumoniae; Salmonella* spp.*; E. coli; Neisseria* spp.;* S. aureus*
Hercik et al. 2017 [33]	Cross-sectional, hospital-based	12	2014/2015	Tanzania	632	≥ 15	114 (18.0%)	*S. aureus*; *K*. *pneumonia*; *P*. *aeruginosa*; *S. pneumonia*; *Moraxella catarrhalis*; Dengue virus; Adenovirus; human Rhinovirus (HRV); *H. influenzae Plasmodium* spp.; *Rickettsia* spp.; *C. burnetii*
Woodruff et al. 1988 [19]	Cross-sectional, hospital-based		1986	South Sudan	130	15–85	69 (53.1%)	Flaviviruses, Alphavirus, Bunyamwera virus, Phlebovirus
Zerfu et al. 2018 [37]	Cross-sectional, community-based	3	2016	Ethiopia	536	> 15	394 (73.5%)	TG *rickettsia; Brucella* spp.
Endale et al. 2020 [40]	Cross-sectional, community-based	7	2018	Ethiopia	281	21–80	154 (54.8%)	Chikungunya virus, Yellow fever virus
Budodo et al. 2020 [39]	Cross-sectional, community-based	3	2020	Tanzania	191	≥ 18	169 (89.6%)	Rift Valley fever virus, Chikungunya virus
Boilat-Blanco et al. 2018 [34]	Cross-sectional, hospital-based	12	2013/2014	Tanzania	519	> 20	56 (11.0%)	Dengue virus; *Plasmodium* spp.; *Salmonella typhi*
Boilat-Blanco et al. 2021 [41]	Cross-sectional, hospital-based	12	2013/2014	Tanzania	519	> 20	56 (11.0%)	Dengue virus; Epstein-Barr virus; West Nile virus; Cytomegalovirus; HIV; Hepatitis B virus; Hepatitis C virus; *S. typhi*; S. *pneumonia*; *Pasmodium* spp.; *Histoplasma capsulatum; C. neoformans; Pneumocystis jirovecii*
Akelew et al. 2022 [42]	Cross-sectional, hospital-based	3	2019	Ethiopia	200	≥ 15	159 (79.5%)	*Plasmodium* spp.; Dengue virus, bacteria (*S. aureus*; *K. pneumoniae*; *Enterobacter cloacae*; TG *Rickettsia*)

### Pooled prevalence of fever of unidentified aetiology

**Fig. 2:**
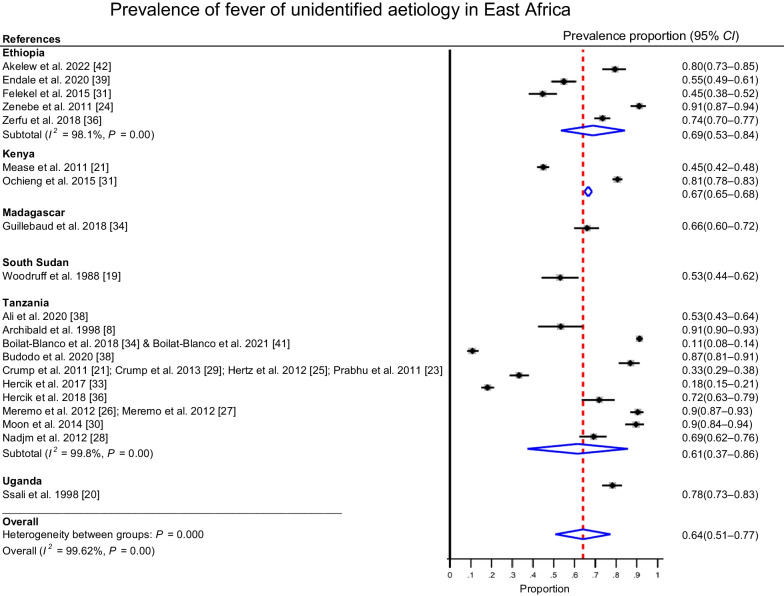
Forest plot showing the pooled estimate of the prevalence of fever with undifferentiated aetiology in East Africa from 20 studies published from 1988 to 2022

The pooled prevalence of fever with unidentified aetiology was 64% (95% *CI*: 51–77%) among adolescent and adult febrile patients seeking health care in East Africa (Fig. 2). Five of the studies had estimates below 50% of which two studies had relatively large *CI*s. High variability was observed among the studies in our meta-analysis, indicated by *I*^2^ = 99.6%.

The analysis by country showed a prevalence of 69% (95% *CI*: 53–84%) in Ethiopia, 67% (95% *CI*: 65–68%) in Kenya and 61% (95% *CI*: 37–86%) in Tanzania. The highest prevalence of fever of unidentified aetiology was observed in Ethiopia, while Tanzania showed the lowest prevalence. The studies from Tanzania showed the highest variability with the largest *CI* compared to the other subgroups of studies. The lowest variability was observed in the studies from Kenya. The studies from Kenya were also focusing on the identification of arboviruses. Studies from Ethiopia focused on isolation of bacterial infections, whereas those from Tanzania demonstrated concomitant identification of bacterial and viral pathogens.

**Fig. 3:**
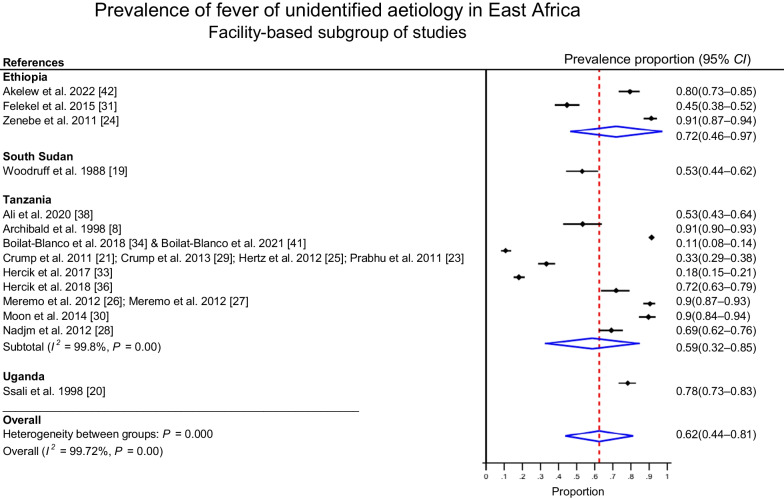
Pooled prevalence of fever with unidentified aetiology in East Africa from sub-group of 13 facility-based studies

Separate analysis of publications from 14 studies conducted at health care facilities showed an overall prevalence of 62% (95% *CI*: 46–97%) of fever of unidentified aetiology. Ethiopian studies (*n* = 5) showed the highest prevalence (72%; 95% *CI*: 46–97%) and Tanzanian studies (*n* = 10) exhibited highest variability (59%; 95% *CI*: 32–85%). The majority of facility-based studies were either conducted in Tanzania (*n* = 7) or in Ethiopia (*n* = 3). Both groups of studies exhibited high variability. Both groups also showed considerable overlap in their group-specific 95% *CI*s. The estimates from the two other studies in Uganda and South Sudan lied within the shared *CI*s (Fig. 3).

### Identified aetiologic agents of febrile illnesses

Overall, the included studies showed a multitude of causative agents of fever in East Africa. The most prevalent pathogen was chikungunya virus (with a prevalence of 17.5% in 6 studies) followed by *Plasmodium* spp. (with a prevalence of 16.4% in 10 studies), *Haemophilus influenzae* (with a prevalence of 14.0% in 2 studies) and dengue fever virus (with a prevalence of 10.1% in 7 studies). *Rickettsia* spp. (with a prevalence of 8.7% in 8 studies) and coagulase negative staphylococci (CoNS) (with a prevalence of 6.5% in 2 studies) were the most prevalent among bacterial zoonotic and bloodstream infections, respectively. Taken together, the included studies identified two main groups of infectious agents; namely, bloodstream infectious and zoonotic pathogens. Out of the six population-based studies, four identified only viral pathogens, whereas the remaining two identified bacterial pathogens. In contrast, all types of pathogens were identified in the health facility-based studies.

#### Bloodstream infectious pathogens

**Fig. 4:**
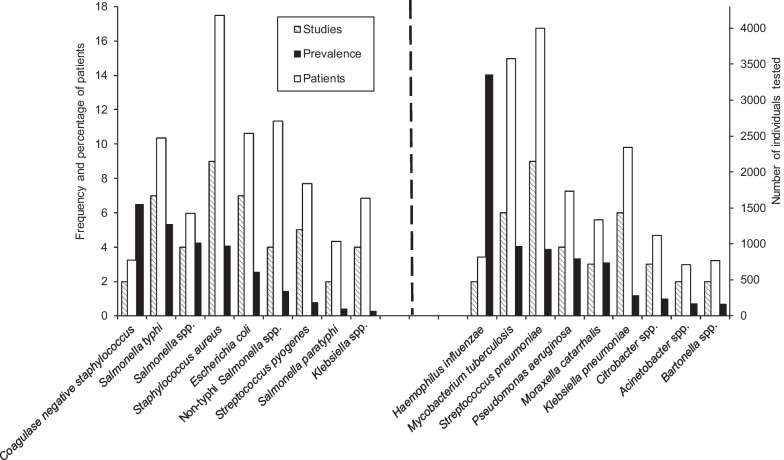
Frequency and percentage of patients (adults and adolescents in East Africa) with reported blood stream pathogens (the pathogens at the right side of the dotted vertical line rarely appear in the blood stream) and number of studies that identified specific pathogens in 20 studies, published from 1988 to 2022

The most frequently reported bloodstream infectious pathogens in the included 20 studies were *Staphylococcus aureus* and *Streptococcus pneumoniae*, each recorded in 9 (45%) studies. Following these were *Salmonella* Typhi and* Escherichia coli*, both recorded in 7 (37%) studies each (Fig. 4). A total of 4176 patients were tested in the studies for *S. aureus* of whom 170 were tested positive owing to a prevalence of 4.1%. The highest prevalence was observed in this group for *H.* *influenza*, reported in two studies and tested positive for 14.0% of 813 individuals.

Apart from *H. influenzae* and CoNS (6.5%), which were the most prevalent pathogens reported in the 20 studies, *Salmonella* Typhi (5.3%; 132/2475), *S. pneumoniae* (4.2%; 170/4003), *Salmonella* spp. (4.2%; 60/1424) and *Mycobacterium tuberculosis* (4.0%; 144/3577) were prevalent bloodstream infections.

#### Zoonotic pathogens

**Fig. 5:**
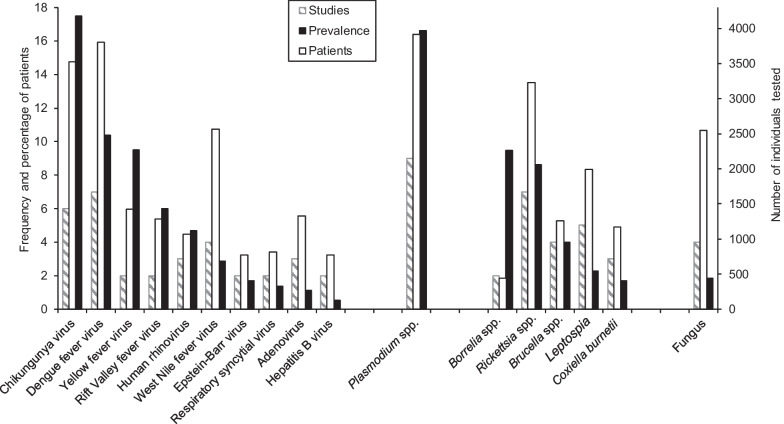
Frequency and percentage of patients (adults and adolescents in East Africa) with reported zoonotic pathogens (viruses, bacteria, protozoan and fungus) and number studies that identified each pathogen in 20 articles, published from 1988 to 2022

Ten viral zoonotic pathogens were reported in the 20 articles analysed (Fig. 5). Chikungunya virus and dengue fever virus were the most prevalent viral pathogens with prevalence of 17.5% and 10.1% in 3529 and 4001 tested patients, respectively. Yellow fever (9.5%) and Rift Valley fever viruses (6.0%) were the next most prevalent pathogens detected. Moreover, West Nile fever virus, human rhinovirus and Epstein-Barr virus were also reported. Furthermore, five bacterial zoonotic pathogens were reported, among which *Rickettsia* spp. [8 studies (40%)] and *Leptospira* spp. [5 studies (25%)] were most frequently reported. Among the other pathogens of this group, the prevalence was highest for *Borrelia* spp. and *Rickettsia* spp.

### Diagnostic techniques used

Various diagnostic methods were applied in the included studies (Table 2). Enzyme-linked immunosorbent assay (ELISA) [in 12 studies (60%)] was the most frequently used technique, most often applying the direct IgM detection. Microscopy, blood culture, rapid diagnostic test (RDT) and PCR were each used in 9 (45%) studies. RDTs were applied for the detection of *Plasmodium*, human immunodeficiency virus (HIV) and dengue virus. In 5 (20%) studies, multiplex PCR analyses were used. Three of these studies (15%) applied TaqMan array cards (TAC) reporting the lowest prevalence of undifferentiated fever. Immunofluorescence assay (IFA) and Western blot were applied in 3 (15%) and 2 (10%) studies, respectively.

**Table 2:** Diagnostic tests used in 20 included studies

Diagnostic method	No. of studies	No. of participants tested	No. of positive cases	Positivity rate (%)
ELISA	12	5030	553	11.0
Microscopy	9	3739	1788	47.8
Blood culture	9	3198	1027	32.1
RDT	9	2901	2137	73.7
PCR	9	2089	1557	74.5

*ELISA* enzyme-linked immunosorbent assay, *IFA* immunofluorescence assay, *PCR* polymerase chain reaction, *RDT* rapid diagnostic test, *No.* Number

## Discussion

In this systematic review and meta-analysis, we found that causes of fever in a large proportion (pooled prevalence = 62%; 95% *CI*: 48–77%) of adolescent and adult febrile patients in East Africa remain unidentified. However, knowledge of pathogens that cause fever is indispensable to inform case management, and hence, there is an urgent need for improved access to diagnostics for patients presenting with a febrile illness. The majority of reviewed studies reported misdiagnoses of febrile cases as “malaria”, thereby underappreciating other causes of fever. Previous studies highlighted important mismatches between clinical diagnosis and case management with confirmed diagnoses [11, 43–47]. This was attributed to the heavy reliance of clinicians on empirical diagnoses, due to limited access to readily available clinical decision support systems and diagnostic tests. Consequently, the burden of disease for various aetiologic agents might be considerably under- or over-estimated. It is important to note that inappropriate and unnecessary use of anti-malarial medications and broad-spectrum antibiotics is a major concern in areas with limited diagnostic capacity [48–51]. For example, a study in Tanzania estimated that approximately 56% of patients with suspected malaria were treated with antimalarials without laboratory confirmation [52]. Similarly, a study in India reported that up to 82% of adult patients with febrile illnesses received antibiotics without microbiological confirmation [53]. A systematic review estimated that up to 69% of antibiotic use in sub-Saharan Africa is inappropriate [54]. Another study showed that 30% of antibiotics used in hospitals were inappropriate in the United States of America [48]. Inappropriate and unnecessary use of antimicrobials can lead to development and spread of antimicrobial resistance (AMR), treatment failure and adverse patient outcomes, unsolicited drug reactions and increased healthcare costs [55]. All of these represent major challenges for global health [56, 57]. Indeed, adequate identification and characterization of potential pathogens as well as increased awareness of clinicians, patients and communities of potential fever aetiologies are crucial to enhance prevention, prevent spread of these pathogens and further enhance early detection and adequate case management.

The included studies revealed common causes of acute febrile illnesses in both hospitalized and ambulatory adolescent and adult patients. Major non-malarial causes of fever were bacterial pathogens (human bloodstream infection), bacterial or viral zoonotic pathogens. Similar findings have been reported recently in another review on non-malarial febrile illnesses with slight differences in the common types of pathogens reported from East Africa. In their review, Elven and colleagues [58] showed a surge of non-typhoidal *Salmonella* spp., while typhoidal *Salmonella* spp. are predominantly reported in our review. The difference might be explained by specific inclusion and exclusion criteria, particularly regarding the age range of individuals included. In our review, we included adolescents and adults (aged ≥ 13 years), while a large proportion of studies reviewed by Elven and colleagues [58] included children. Despite this difference in age profiles, there was a similar viral distribution in the two studies. A narrative review pertaining to the epidemiology of febrile illnesses in sub-Saharan Africa reported observations similar to our findings [11]. Indeed, Maze and colleagues [11] reported that pathogens isolated from hospitalized patients were more likely to be bloodstream infections, while common causes of fever among ambulatory patients were due to arboviruses and other respiratory pathogens [44].

Evidence of exposure to, and infection by, these common agents suggests their potential endemicity in East Africa, and hence, highlighting the clinical importance of the necessity of preventive measures targeting these pathogens and of the implementation of improved diagnostic techniques for their timely and reliable detection and effective treatment. Integrated approaches of pathogen detection, including setting-specific surveillance-response systems and application of multiple-pathogen detection technologies are, therefore, of paramount importance [59].

Collation of high-quality data is central to setting up effective surveillance-response systems. Yet, in East Africa, resources are often limited [60]. Health system strengthening to enhance surveillance-response activities will increase the potential capacity to detect causative agents in East Africa [38]. In this regard, establishing comprehensive fever syndromic surveillance-response approaches [61], with improved local availability of inexpensive, rapid, reliable and integrated diagnostic techniques that are suitable for point-of-care (POC), multi-pathogen detection using single-sample would lead to considerable impact by supporting health care professionals to offer more accurate and certain diagnosis and management, which would improve patient outcomes. Pilot studies employing a metagenomics approach are warranted and should determine costs, feasibility and scalability [62, 63].

### Implications of our findings

The findings of this review suggest that a large share of febrile adolescents and adults in East Africa experience inappropriate care by either receiving unnecessary or ineffective medications or being withheld from essential medications. Hence, while diagnostic capacity of East African countries is limited due to lack of human and financial resources, health systems strengthening and integrated approaches for detecting pathogens will support improved detection of aetiologic agents of febrile illnesses in East Africa. In turn, this will improve febrile case management, inform clinical epidemiology, refine understanding of the endemic disease profiles and thus positively impact on the health and well-being of the affected communities.

### Recommendations for clinicians, hospital and public health communities

Based on the findings of this study, there are a couple of specific recommendations that can be drawn for policy and practice in East Africa. First, the study suggests that a considerable proportion of fevers in East Africa is of unknown or unidentified aetiology. The high prevalence of fever of unidentified aetiology indicates that there are significant gaps in diagnostic capabilities and surveillance systems, particularly in remote rural areas. Strengthening diagnostic capacity and surveillance-response systems for fever of unidentified aetiology could lead to more accurate diagnoses and more effective treatment, as well as more timely detection and control of febrile illnesses. This could include the use of multiplex PCR technologies, which have shown promise in identifying a wider range of pathogens compared to traditional diagnostic methods. Second, the study emphasises the importance of continued investment in public health initiatives aimed at preventing the transmission of arboviral diseases and reducing the burden of bacterial infections, which were identified as the most common causes of febrile illnesses in our analysis. Third, our study suggests the need for increased collaboration and coordination among public health authorities and healthcare providers in the region. This could involve the development of regional networks for surveillance, diagnosis and treatment of fever of unidentified aetiology, as well as the sharing of best practices and resources among healthcare providers. By working together to improve diagnostic capacity and strengthen surveillance-response systems, policy-makers and practitioners in East Africa could make significant progress towards reducing the burden of fever of unidentified aetiology in the region.

### Strengths and limitations

In this review, we did not restrict our search terms to capture the keywords that show up in the title or abstract of published articles only. Consequently, a very large number of hits resulted from our initial search (> 14,000). We also hand-searched references of included studies to identify potential additional documents not identified by our electronic search. Furthermore, to increase the robustness of the review, we extracted information from included studies by strictly following a systematic procedure. However, a limitation of this review is that we did not perform a search of the grey literature (literature produced outside of the indexed databases, such as government reports, policy statements, pre-prints, etc.). In addition, the search strategy may have missed studies published in local journals that are not indexed in the selected databases. Likewise, the final set of studies that met our inclusion criteria were all published in English, though it is unlikely that this biased our analysis, since we did not apply any language restriction. Moreover, although some of the studies provided supplementary materials detailing the tests performed and the number and types of pathogens tested per patient, most of the included studies did not publish this fine-grained level of detail. Hence, our findings should be considered with some caution, as we could only capture the full range of pathogens reported in all included studies. Also, due to the limited data available, the findings were not further stratified by quality of diagnostic evidence. Finally, it is important to acknowledge that meta-analyses are inevitably constrained by the quality and diversity of the available data. As a result, despite our efforts to address heterogeneity, some degree of residual heterogeneity may remain.

## Conclusions

Febrile patients, both ambulatory and hospitalised, require appropriate diagnosis to receive adequate management and therapy. This systematic review and meta-analysis provides new evidence that causes of fever in a large proportion (over 60%) of febrile adolescent and adult patients in East Africa remain unidentified. The outcome of this meta-analysis and the results of the individual studies reviewed support the notion that the majority of febrile patients attending health care facilities experience inappropriate care by either receiving unnecessary medications or being withheld from essential effective ones. Hence, we call for increased awareness of health professionals and policy-makers, improved access and availability of affordable and accurate diagnostic tests and the use of integrated approaches of multi-pathogen detection. Together, such a package holds high potential to improve patient outcomes in East Africa and elsewhere in LMICs.

## Supplementary Information

**Additional file 1: Table S1.** Summary of quality assessment of included studies by the Joanna Briggs Institute critical appraisal tool assessment.

## Data Availability

All data generated or analysed during this study are included in this published article and its additional information files.

## Abbreviations

AMR: Antimicrobial resistance
AU: African Union
AUF: Acute undifferentiated fever
CI: Confidence interval
CINAHL: Cumulative Index to Nursing & Allied Health Literature
CoNS: Coagulase negative staphylococci
DALYs: Disability-adjusted life years
ELISA: Enzyme-linked immunosorbent assay
FUO: Fever of unknown origin
FWAS: Fever without apparent source
FWS: Fever without source
HIV: Human immunodeficiency virus
IFA: Immunofluorescence assay
JBI: Joana Briggs Institute
LMICs: Low- and middle-income countries
PCR: Polymerase chain reaction
POC: Point-of-care
PRISMA: Preferred Reporting Items for Systematic Reviews and Meta-Analyses
RDT: Rapid diagnostic test
TAC: TaqMan array cards
UDF: Undifferentiated fever
