# Supplementary material for: Prevalence of fever of unidentified aetiology in East African adolescents and adults: a systematic review and meta-analysis
Source: Infect Dis Poverty. 2023 May 25;12:55. doi: 10.1186/s40249-023-01105-z (PMC10210420; doi:10.1186/s40249-023-01105-z)
Supplement: Supplementary file 1 — Additional file 1: Table S1. Summary of quality assessment of included studies by the Joanna Briggs Institute critical appraisal tool assessment. [file 40249_2023_1105_MOESM1_ESM.docx]

| **S/N** | **Reference** | **Quality of the study (certainty of evidence)** | | | | | | | | | | |
| --- | --- | --- | --- | --- | --- | --- | --- | --- | --- | --- | --- | --- |
|  |  | Was the sample frame appropriate to address the target population? | Were study participants sampled in an appropriate way? | Was the sample size adequate? | Were the study subjects and the setting described in detail? | Was the data analysis conducted with sufficient coverage of the identified sample? | Were valid methods used for the identification of the condition? | Was the condition measured in a standard, reliable way for all participants? | Were appropriate statistical analyses applied? | Was the response rate adequate, and if not, was the low response rate managed appropriately? | **Overall appraisal** | **Comments** |
| 1 | Ali et al. 2020 (38) | Yes | Yes | Yes | Yes | Yes | Yes | Yes | Yes | Yes | Included |  |
| 2 | Archibald et al. 1998 (8) | Yes | Yes | Yes | Yes | Yes | Yes | Yes | Yes | Yes | Included |  |
| 3 | Boillat-Blanco et al. 2021 (41) | Yes | Yes | Yes | Yes | Yes | Yes | Yes | Yes | Yes | Included |  |
| 4 | Boillat-Blanco et al. 2018 (34) | Yes | Yes | Yes | Yes | Yes | Yes | Yes | Yes | Yes | Included |  |
| 5 | Boone et al. 2017 (43) | Yes | Yes | Yes | Yes | Yes | Yes | Yes | Yes | Yes | Excluded | Results for adults and children were not presented separately |
| 6 | Budodo et al. 2020 (39) | Yes | Yes | Yes | Yes | Yes | Yes | Yes | Yes | Yes | Included |  |
| 7 | Crump et al. 2011 (21) | Yes | Yes | Yes | Yes | Yes | Yes | Yes | Yes | Yes | Included |  |
| 8 | Crump et al. 2013 (29) | Yes | Yes | Yes | Yes | Yes | Yes | Yes | Yes | Yes | Included |  |
| 9 | Endale et al. 2020 (40) | Yes | Yes | Yes | Yes | Yes | Yes | Yes | Yes | Yes | Included |  |
| 10 | Felekel et al. 2015 (31) | Yes | Yes | Yes | Yes | Yes | Yes | Yes | Yes | Yes | Included | Separate results were presented for adults |
| 11 | Grossi-Soyster et al. 2017 (44) | Yes | Yes | Yes | Yes | Yes | Yes | Yes | Yes | Yes | Excluded | Separate results were not presented for febrile patients |
| 12 | Guillebaud et al. 2018 (35) | Yes | Yes | Yes | Yes | Yes | Yes | Yes | Yes | Yes | Included |  |
| 13 | Hercik et al. 2017 (33) | Yes | Yes | Yes | Yes | Yes | Yes | Yes | Yes | Yes | Included |  |
| 14 | Hercik et al. 2018 (36) | Yes | Yes | No | Yes | Yes | Yes | Yes | Yes | Yes | Included |  |
| 15 | Hertz et al. 2012 (25) | Yes | Yes | Yes | Yes | Yes | Yes | Yes | Yes | Yes | Included |  |
| 16 | Kajeguka et al. 2016 (45) | Yes | Yes | Yes | No | Yes | Unclear | Yes | Yes | Unclear | Excluded | Results for adults and children were not presented separately |
| 17 | McArthy et al. 1996 (46) | Yes | Yes | Not clear | Yes | Yes | Yes | Yes | Yes | Not clear | Excluded | Results for adults and children were not presented separately |
| 18 | Mease et al. 2011 (22) | Yes | Yes | Yes | Yes | Yes | Yes | Yes | Yes | Yes | Included |  |
| 19 | Meremo et al. 2012 (26) | Yes | Yes | Yes | Yes | Yes | Yes | Yes | Yes | Yes | Included |  |
| 20 | Meremo et al. 2012 (27) | Yes | Yes | Yes | Yes | Yes | Yes | Yes | Yes | Yes | Included |  |
| 21 | Moon et al. 2014 (30) | Yes | Yes | Yes | Yes | Yes | Yes | Yes | Yes | Yes | Included |  |
| 22 | Nadjm et al. 2012 (28) | Yes | Yes | No | Yes | Yes | Yes | Yes | Yes | Yes | Included |  |
| 23 | Nyataya et al. 2020 (47) | Yes | Yes | Yes | Yes | Yes | Yes | Yes | Yes | Yes | Excluded | Results for adults and children were not presented separately |
| 24 | Ochieng et al. 2016 (32) | Yes | Yes | Yes | Yes | Yes | Yes | Yes | Yes | Yes | Included |  |
| 25 | Prabhu et al. 2011 (23) | Yes | Yes | Yes | Yes | Yes | Yes | Yes | Yes | Yes | Included |  |
| 26 | Rubach et al. 2015 (48) | Yes | Yes | Yes | Yes | No | Yes | Yes | Yes | No | Excluded | Focused on febrile patients with highly severe conditions |
| 27 | Salah et al. 1988 (49) | Yes | Not clear | No | Yes | Yes | Yes | Yes | Yes | Not clear | Excluded | Only 41 of the participants were febrile and separate analyses for febrile patients were not conducted |
| 28 | Ssali et al. 1998 (20) | Yes | Yes | Yes | Yes | Yes | Yes | Yes | Yes | Yes | Included |  |
| 29 | Thiga et al. 2015 (50) | Yes | Not clear | Yes | Yes | No | Yes | Yes | Yes | Yes | Excluded | Results for adults and children were not presented separately |
| 30 | Tigoi et al. 2015 (51) | Yes | Not clear | Yes | Yes | Not clear | Yes | Yes | Yes | Not clear | Excluded | The overall number of febrile patients seen during the data collection was not mentioned; 10 individuals of unknown age; - analysis did not separate children from adults/adolescents |
| 31 | Wasihun et al. 2015 (52) | Yes | Yes | Yes | Yes | Yes | Yes | Yes | Yes | Yes | Excluded | Results for adults and children were not presented separately |
| 32 | Woodruff et al. 1988 (19) | Yes | Not clear | No | Yes | Yes | Yes | Yes | Yes | Yes | Included |  |
| 33 | Zenebe et al. 2011 (24) | Yes | Yes | Yes | Yes | Yes | Yes | Yes | Yes | Yes | Included |  |
| 34 | Zerfu et al. 2018 (37) | Yes | Yes | Yes | Yes | Yes | Yes | Yes | Yes | Yes | Included |  |
| 35 | Akelew et al. 2022 (42) | Yes | Yes | Yes | Yes | Yes | Yes | Yes | Yes | Yes | Included |  |
